# Screening Spring Wheat Genotypes for *TaDreb-B1* and *Fehw3* Genes under Severe Drought Stress at the Germination Stage Using KASP Technology

**DOI:** 10.3390/genes14020373

**Published:** 2023-01-31

**Authors:** Elsayed A. Mohamed, Asmaa A. M. Ahmed, Matías Schierenbeck, Mohamed Y. Hussein, P. Stephen Baenziger, Andreas Börner, Ahmed Sallam

**Affiliations:** 1 Department of Genetics, Faculty of Agriculture, Assiut University, Assiut 71526, Egypt; 2 Resources Genetics and Reproduction, Department Genebank, Leibniz Institute of Plant Genetics and Crop Plant Research (IPK), Corrensstr. 3, OT Gatersleben, D-06466 Stadt Seeland, Germany; 3 CONICET CCT La Plata, 8 N°1467, La Plata 1900, Argentina; 4 Department of Agronomy and Horticulture, University of Nebraska–Lincoln, Lincoln, NE 68583, USA

**Keywords:** water deficit, genetic variation, germination traits, *Triticum aestivum* L., PEG, KASP, *TaDreb-B1* and *Fehw3* genes

## Abstract

Drought stress is a major yield-limiting factor throughout the world in wheat (*Triticum aestivum* L.), causing losses of up to 80% of the total yield. The identification of factors affecting drought stress tolerance in the seedling stage is especially important to increase adaptation and accelerate the grain yield potential. In the current study, 41 spring wheat genotypes were tested for their tolerance to drought at the germination stage under two different polyethylene glycol concentrations (PEG) of 25% and 30%. For this purpose, twenty seedlings from each genotype were evaluated in triplicate with a randomized complete block design (RCBD) in a controlled growth chamber. The following nine parameters were recorded: germination pace (GP), germination percentage (G%), number of roots (NR), shoot length (SL), root length (RL), shoot–root length ratio (SRR), fresh biomass weight (FBW), dry biomass weight (DBW), and water content (WC). An analysis of variance (ANOVA) revealed highly significant differences (*p* < 0.01) among the genotypes, treatments (PEG25%, PEG30%) and genotypes × treatment interaction, for all traits. The broad-sense heritability (H^2^) estimates were very high in both concentrations. They ranged from 89.4 to 98.9% under PEG25% and from 70.8 to 98.7% under PEG30%. Citr15314 (Afghanistan) was among the best performing genotypes under both concentrations for most of the germination traits. Two KASP markers for *TaDreb-B1* and *Fehw3* genes were used to screen all genotypes and to study the effect of these on drought tolerance at the germination stage. All genotypes with *Fehw3* (only) showed a better performance for most traits under both concentrations compared to other genotypes having *TaDreb-B1* or having both genes. To our knowledge, this work is the first report showing the effect of the two genes on germination traits under severe drought stress conditions.

## 1. Introduction

Germination and seedling growth are considered one of the critical stages for the growth and development of wheat (*Triticum aestivum* L.) [1,2,3]. Basically, if the crop does not germinate or if the seedling growth is poor, the wheat yield can be greatly reduced. In the context of global warming, increased early drought stress is expected in the coming decades, which means the production of this staple crop in many regions of the world will be more vulnerable [4]. It has been widely documented that the basis of drought tolerance is particularly complex and involves many genetic, morphological and physiological components [5]. Water shortage during early stages interferes with cellular activity and induces numerous metabolic changes that reduce the photosynthetic activity and respiration rate of seedlings, leading to decreased leaf area expansion and subsequent effects on biomass generation and grain yield [6,7]. Thus, identifying traits that contribute to drought stress tolerance and the molecular location of the genetic factors that determine different growth parameters in wheat breeding is a research priority to accelerate the rate of genetic gain.

Early growth stages, such as germination and seedlings, have been reported to be extremely sensitive to drought stress [3,8]. Improving wheat’s drought tolerance through breeding at the germination stage is particularly important, as this growth stage affects all subsequent growth stages. Therefore, obtaining wheat cultivars that are highly tolerant to drought stress is urgent. The analysis of plant growth in a simulated environment can help to identify tolerance-related traits by reducing environmental noise (variation) associated with field experiments [1]. High molecular mass polyethylene glycol (PEG-6000) has been widely used to induce osmotic stress in controlled conditions [9,10,11,12]. The response to PEG is similar to that induced by drought, causing a reduction in germination, seedling vigor, and overall growth [2,8,13]. In wheat PEG concentrations in the range of 5%–15% [14] have been frequently evaluated. However, studies reporting its effect at concentrations greater than 25%, that mimic severe drought stress are lacking.

Advances in deoxyribonucleic acid (DNA) markers should be utilized to accelerate the breeding program to improve drought tolerance. DNA markers associated with known drought genes can be useful to rapidly screen numerous genotypes at a lower cost and less labor. Dehydration responsive element binding protein (*TaDreb-B1*) genes are some of the most vital regulators of complex drought stress genetic networks in wheat. The high expression of these genes has been associated with adaptation to drought stress in this crop [15]. Moreover, Fructan 1-exohydrolase w3 (*Fehw3*) gene contribute to the high levels of stem water-soluble carbohydrate remobilization under terminal drought stress [4]. Several authors have reported that *TaDreb-B1* and *Fehw3* genes are involved in abiotic stress tolerance parameters in several crops [8,16,17,18]. Chen et al. [19] reported that wheat accessions, carrying *TaDreb-B1* haplotypes1 and 3, showed drought tolerance. Moreover, Yang et al. [20] reported that *TaDreb-B1* genes improved drought and frost tolerance in barley and wheat seedlings.

Many specific markers (e.g., SSR) were designed for drought genes, such as *TaDreb-B1* and *Fehw3*, and which were used for screening wheat genotypes. However, sometimes the reproducibility is low and sensitive to lab conditions. Instead, competitive allele specific polymerase chain reaction (PCR) (KASP) technology is a flexible genotyping platform that is safe and cost-effective. KASP markers are designed to target the SNP allele of a gene, providing extremely accurate genotyping and reliable testing for the presence of target genes [17]. The KASP markers should be tested and validated in different genetic backgrounds before their use in marker-assisted selection. Thus, screening the selected tolerant genotypes carrying both genes, *TaDreb-B1* and *Fehw3*, using DNA molecular markers could be considered a key tool for selecting parents for future crossing in breeding programs and improving lines among the progeny of those crosses.

The objectives of the current study were to (1) assess the germination performance and phenotypic variation among 41 highly diverse spring wheat genotypes, selected from Ahmed et al. [21]; under severe drought stress, (2) screen the presence of specific drought genes via KASP technology (*TaDreb-B1* and *Fehw3*), (3) test the marker-traits association between the two KASP markers and germination traits under drought stress.

## 2. Materials and Methods

### 2.1. Plant Material

The plant material consisted of 41 diverse spring wheat genotypes (Supplementary Table S1) highly adapted to Egyptian conditions (Ahmed Sallam, personal communication). The 41 genotypes representing tolerance, intermediate tolerance and susceptibility to drought at seedling stage and were selected based on drought tolerance index (DTI) estimated by Ahmed et al. [21]. The genotypes were collected from the U.S. National Plant Germplasm (United States Department of Agriculture, USA). Supplementary Figure S1 shows the number of genotypes used from each country. The largest number of genotypes was from the continent of Africa (21 genotypes). Out of the 41 genotypes, Egypt had the highest number of tested genotypes (nine accessions).

### 2.2. Experimental Layout

The experimental layout applied was based on the protocol suggested by Thabet et al. [22] and Haseeb et al. [23]. Experiments were conducted in the Plant Genetics Lab, Faculty of Agriculture (Assiut University, Egypt). Genotypes were tested for their tolerance to drought at the germination stage under two different concentrations of PEG (25% and 30%). Twenty seedlings from each genotype were evaluated under drought stress in this stage. The genotypes were sown in triplicate (replications) with a randomized complete block design (RCBD) in a controlled growth chamber. The seeds were placed in a Petri dish containing filter paper with a 9 cm diameter and irrigated with 10 ml of the corresponding solution. The dishes were placed in an incubator under 22 °C in the darkness. The seed was considered germinated and counted when the radicle reached 2 mm in length. Germination parameters were scored according to International Seed Testing Association rules [24]. At the end of the experiment (on the 13th day), the seedling-related parameters were manually scored on 10 plants of each tested genotype.

### 2.3. Traits Scoring

Germination percentage (*G*%) was scored daily (every 24 h) during the experiment for up to 12 consecutive days to calculate the daily *G*% for each genotype.

(1) $$G\%=\frac{n}{N}\times 100$$

where *n* is the number of germinated seeds at the end of the experiment (on the 13th day) and *N* is the total number of seeds sown = 20 seeds.

Germination pace (*GP*): was calculated using the following equation:

(2) $$GP=\frac{N}{\sum \left(n\times g\right)}\times 100$$

where *N* is the total number of germinated seeds at the end of the experiment, and *n* is the number of newly germinated seeds on a certain day (*g*), *g* = (1, 2, 3, ...etc.).

At the end of the experiment (on the 13th day) the seedling traits were recorded as follows:

1. Number of roots (NR) was scored on the last day of the experiment to determine the NR for each germinated genotype.
2. Root length (RL) was manually measured using a scaled or graduated ruler (cm) from the bottom of the seed tip to the end of the root.
3. Shoot length (SL) was manually measured using a scaled or graduated ruler (cm) from the top of the seed tip to the end of the shoot.
4. Shoot–root length ratio (SRR) was calculated as the ratio for each genotype by dividing SL by RL.
5. Fresh biomass weight (*FBW*) was scored by weighing (g) germinated seeds (including root and shoot) using a balance (0.0001 g—Sartorius AC 1215, Germany)
6. Dry biomass weight (*DBW*) was estimated by drying the germinated seeds in an aerated oven at 70 °C for 72 h and then measuring their weight (g). The fresh and dry biomass weight for each genotype under both treatments was used for assessing the water content.
7. Water content (*WC*) was calculated by:

(3) $$WC=\frac{FBW-DBW}{FBW}\times 100$$

Moreover, the same genotypes were previously evaluated for drought tolerance at the seedling stage by Ahmed et al. [21], who created the drought tolerance index (DTI) for each genotype. The detailed calculations of the DTI were extensively explained in Ahmed et al. [21]. In this study, we used the DTI values of the same genotypes to study the correlation between (DTI) calculated and all traits scored at the germination stage. 

### 2.4. Statistical Analysis of Phenotypic Data

The statistical analysis of all phenotypic data was carried out for estimating variance and covariance using PLABSTAT software [25]. Two analyses of variances (ANOVA) were calculated. The first ANOVA was calculated for all traits under each treatment using the following statistical model:

*Yij* = *µ* + *g*_*i*_ + *r*_*j*_ + *gr*_*ij*_ (*error*) (4)

where *Yij* is the observation of genotype *i* in replication *j*, *µ* is the general average, *gi* and *rj* are the main effects of genotypes and replication, respectively, and *gr_ij_ (error)* is the interaction between genotype *i* and replication *j*.

In the second ANOVA, the two treatments (PEG25% and PEG30%) were included to see the variation between and within each treatment using the following model:

*Y*_*ijk*_ = *µ* + *g*_*i*_ + *r*_*j*_ + *t*_*k*_ + *tg*_*ik*_ + *tgr*_*ijk*_ (5)

where *Y_ijk_* is the observation of genotype *i* in replication *j.* In treatment *k, μ* is the general mean; *g_i_*, *r_j_*, and *t_k_* are the main effects of the genotypes, replications, and treatments, respectively. *tg_ik_* is the genotype × treatment interaction. *tgr_ijk_* is the genotype × replications × treatment interaction (error). Genotypes and replications were considered fixed and random effects, respectively. Treatments were considered fixed effects in the ANOVA analysis.

Broad-sense repeatability (*H*^2^) estimates for each trait were calculated by PLABSTAT:

(6) $$\;\;\;\;\;\;\;\;\;\;\;\;\;\;\;\;\;\;\;\;\;\;\;\;\;\;\;\;\;\;H^{2}=\frac{\sigma_{G}^{2}}{\sigma_{G}^{2}+\left(\sigma_{GR}^{2}\right)}$$

where *G* refers to genotypic variation and *r* refers to the phenotypic variation. The Spearman’s rank correlation coefficient was imputed by PLABSTAT to estimate the phenotypic correlation between the traits. Microsoft Office Excel 2010 and R software [26] were used to create some graphical presentations of the results of the analysis. Correlations among the traits were plotted using MVApp v2.0 [27].

The reduction in each trait due to severe drought stress (*PEG*30%) was calculated for all germination traits that were scored in this study, based on the average of each trait and using the following equation:

(7) $$RDD-trait=\left(\frac{X_{PEG25\%}-X_{PEG30\%}}{X_{25\%}}\right)\times 100$$

where *X_PEG_*_25%_ and *X_PEG_*_30%_ are the means of a trait for each genotype under concentration 25% and concentration 30% of PEG, respectively.

### 2.5. DNA Extraction and KASP Genotyping

DNA was extracted from the leaves of 41 genotypes using the BioSprint 96 automatic DNA extractor. The DNA concentrations were diluted at 50 ng/µL in sterile distilled water to be used in the KASP-SNP PCR reaction. All samples were arrayed in 96 well plates. A 10 µL reaction with 5 µL of DNA from each sample and 5 µL of the KASP reaction mix, including 0.14 µL of the *Fhb1* assay mix from LGC-Genomics (Middlesex, UK), was used. 

Two KASP markers for *TaDreb-B1* and *Fehw3* genes were ordered from LGC-Genomics (Middlesex, UK) (Table 1). Thermal cycling conditions lasted 15 min at 94 °C. This was followed by 10 cycles of touchdown PCR as follows: 94 °C for 20 s and 65–57 °C for 60 s (dropping to 0.8 °C per cycle). This was followed by 26 cycles of regular PCR as follows: 94 °C for 20 s and 55 °C for 60 s. The plate of samples was read via FLUOstar Omega fluorescence. To determine the absence or presence of the *TaDreb-B1* and *Fehw3* genes, the target allele (presence of gene) was labelled with FAM (blue), while the non-target allele A (absence of gene) was labelled with HEX (red). The analysis of KASP-PCR products was performed using Klustercaller v2.22.0.5 software (LGC, Biosearch Technologies, Beverly, MA, USA). SNPviewer software (LGC, Biosearch Technologies, Beverly, MA, USA) was used for SNP allele calling. Single-marker analysis (SMA) was performed to test the marker-trait association between the two KASP markers and all germination traits scored under the two treatments as described by Mourad et al. [28].

## 3. Results

### 3.1. Genetic Variation in Drought Tolerance at the Germination Stage

A set of 41 spring wheat phenotypes was tested in the germination stage under two different concentrations of PEG-6000 (25% and 30%). Table 2 presents the analysis of variance (ANOVA) for all germination traits scored under the two treatments. The results of the combined ANOVA revealed highly significant differences (*p* < 0.01) among the genotypes in all traits. Highly significant differences between the two concentrations of PEG were detected for the nine traits evaluated. The differences among the replications were only significant in GP and DBW traits, while the treatment × genotypes interaction was highly significant for all traits (Table 2). The broad-sense heritability (H^2^) estimates ranged from 70.8 for SRR to 98.9 for DBW presence. The maximum (Max), minimum (Min), mean, and *F-value* among the genotypes in each treatment is presented in Table 3. The H^2^ estimates for all traits scored were high in both concentrations (89.4% to 98.9% for PEG25% and 70.8% to 98.7% for PEG30%) (Table 3).

**Figure 1:**
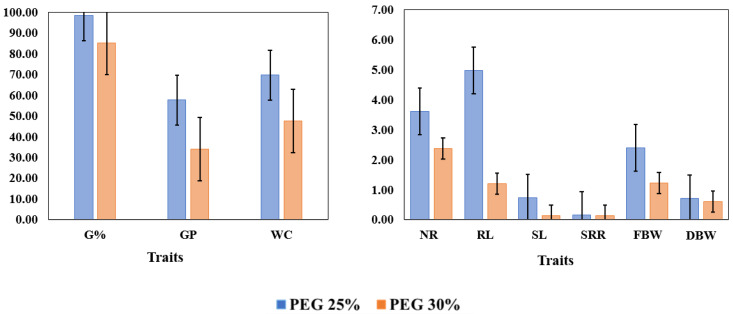
Mean values for germination and seedling growth parameters under PEG-6000 concentrations (PEG25%, PEG30%) among 41 spring wheat genotypes; Germination percentage (G%); Germination pace (GP); Water content (WC); Number of root (NR); Root length (RL); Shoot length (SL); Shoot-root length ratio (SRR); Fresh biomass weight (FBW); Dry biomass weight (DBW).

It was observed that all tested genotypes had a large phenotypic variation under both concentrations (25% and 30%) (Supplementary Figure S2a–c). The phenotypic scores for each tested genotype for germination traits scored under PEG25% and PEG30% are shown in Supplementary Tables S2a,b and S3a,b. The means of all traits reduced significantly under PEG30% compared to their values under PEG25% (Figure 1). On the other hand, it was noted that the genetic variation (*F-value*) among the tested genotypes in all scored traits under PEG30% was higher compared to those detected for PEG25%. Highly significant reductions in all traits due to severe drought stress (PEG30%) were detected as follows when PEG30% and PEG25% were compared: SL (−81.1%), RL (−75.9%), FBW (−48.9%), GP (−41.1%), NR (−34.3%), and WC (−31.6%) (Supplementary Table S4 and Figure 1). The lowest reductions were detected for DBW (−15.5%), G% (−13.4%) and SRR (−6.7%), respectively, (Supplementary Table S4).

### 3.2. Correlation among Germination and Seedling Growth Parameters

**Figure 2:**
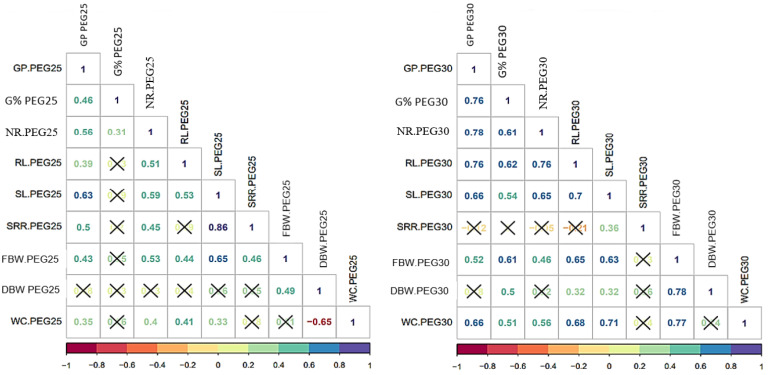
Correlations between germination and seedling growth parameters under PEG25% (**left**) and PEG30% (**right**) among 41 spring wheat genotypes. Germination pace (GP); Germination percentage (G%); Number of root (NR); Root length (RL); Shoot length (SL); Shoot-root length ratio (SRR); Fresh biomass weight (FBW); Dry biomass weight (DBW); Water content (WC); where (X) stands for non-significant correlations.

Highly significant and positive associations were detected among the majority of the characters evaluated in both PEG treatments. SRR and DBW showed weaker associations (Figure 2). In general terms, higher correlations were reported under PEG30% treatments for GP, G%, NR, RL, SL, FBW, and WC compared to PEG25%. Some discrepancies between correlations among the evaluated characters were detected under both PEG treatments.

Under PEG25% treatments, the germination pace (GP) showed highly significant correlations with G% (0.46 **), NR (0.56 **), RL (0.39 **), SL (0.63 **), SRR (0.50 **), FBW (0.43 **), and WC (0.35 *). For their part, under PEG30% treatments, higher correlations were reported between GP with: G% (0.76 **), NR (0.78 **), RL (0.76 **), SL (0.66 **), FBW (0.52 **), and WC (0.66 **). The germination percentage (%) under PEG25% treatments only showed significant correlations with NR (0.31 *). In contrast, under PEG30% treatments, highly significant associations were detected among G% and NR (0.61 **), RL (0.62 **), SL (0.54 **), FBW (0.61 **), DBW (0.50 **), and WC (0.51 **) (Figure 2).

The number of roots (NR) was positively correlated with RL (0.51 **), SL (0.59 **), SRR (0.45 **), FBW (0.53 **), and WC (0.40 *) under PEG25% treatments, while under PEG30%, this trait showed values of RL (0.76 **), SL (0,65 **), FBW (0.46 **), and WC (0.56 **). Under PEG25% concentration, the root length (RL) showed positive correlations with SL (0.53 **), FBW (0.44 **), and WC (0.41 **), while more significant correlations were documented under PEG30% treatments for this trait and SL (0.70 **), FBW (0.65 **), DBW (0.32 *), and WC (0.68 **). Moreover, a positive correlation existed between SL with SRR (0.86 **), FBW (0.65 **), and WC (0.33 *) under PEG25% drought conditions, while generally lower correlations of SRR (0.36 **), FBW (0.63 **), DBW (0.32 *), and WC (0.71 **) were reported under PEG30% drought conditions (Figure 2). The shoot-root length ratio (SRR) showed a positive correlation with FBW (0.46 **) under PEG25% stress, while no other significant correlations existed between SRR and other traits under PEG30% treatments, except SL (0.36 **).

The fresh biomass weight (FBW) was positively correlated with DBW (0.49 **) under PEG25% treatments. Correlations between FBW and other traits were generally higher under PEG30% with DBW (0.78 **) and WC (0.77 **). Dry biomass weight (DBW) under PEG25% only showed positive associations with FBW (0.49 **) and negative significant correlations with WC (−0.65 **) and DTI (−0.549 **). Under PEG30% treatments, a positive correlation was found only between DBW and FBW (0.78 **) and a negative and significant association with DTI (−0.406 **) (Figure 2 and Table 4). 

Few significant correlations were detected when germination and growth traits were plotted with DTI (Table 4). Negative, highly significant correlations were detected between DTI with FBW under both PEG concentrations (−0.350 * for PEG25% and −0.396 * for PEG30%) and DBW (−0.549 ** for PEG25% and −0.406 ** for PEG30%) under both concentrations.

Moreover, correlations of the same traits between PEG treatments (PEG25 % and PEG 30%) were also studied and the response of the genotypes was evaluated (Supplementary Figure S3a–i). The G% (r = 0.21) and GP (r = 0.52 **) showed a positive correlation when both concentrations were tested. Genotypes Citr15314, Flcol-4408, Iwa8600064, Hmira, Sids-12, Gimmeiza-12, and Rhodesian Sabanero presented the best G% performance under both drought stress conditions (Supplementary Figure S3a). Accessions Citr15314, Iwa8600064 and pirotriks-28 showed higher values for GP (Supplementary Figure S3b). The RL (0.28) and NR (0.49 **) also presented positive correlations when both concentrations were plotted. The genotypes Citr15314, Sakha-93, and Iwa8600064 showed the best performance under both drought conditions in RL (Supplementary Figure S3c). The Citr15314, Iwa8600064, and Grekum-105 genotypes showed the highest performance under both concentrations in NR (Supplementary Figure S3d). The SL (0.24) and SRR (0.23) showed a positive correlation under both concentrations tested. Genotypes Citr15314, Misr-1, and Rhodesian Sabanero had the highest performance in SL (Supplementary Figure S3e), while Mg 27959, PI525221, and Shandweel-1 showed the higher value in SRR under both 25% and 30% of PEG (Supplementary Figure S3f). The FBW (0.367 *) and DBW (0.393 *) also showed positive correlations under both PEG treatments, with PI525434, Sakha-93, and Gimmeiza-12 showing a good performance in both variables and concentrations (Supplementary Figure S3g). The following genotypes PI525434, PI154279, and Shandweel-1 had the highest values in DBW (Supplementary Figure S3h). WC had a positive correlation under both concentrations of PEG (0.067), and genotypes Grekum-105, Citr15314, and PI238391 showed the best performance under the two used concentrations of PEG used (Supplementary Figure S3i).

### 3.3. The Effect of TaDreb-B1 and Fehw3 via KASP Technology on Germination Traits under Drought Stress

**Figure 3:**
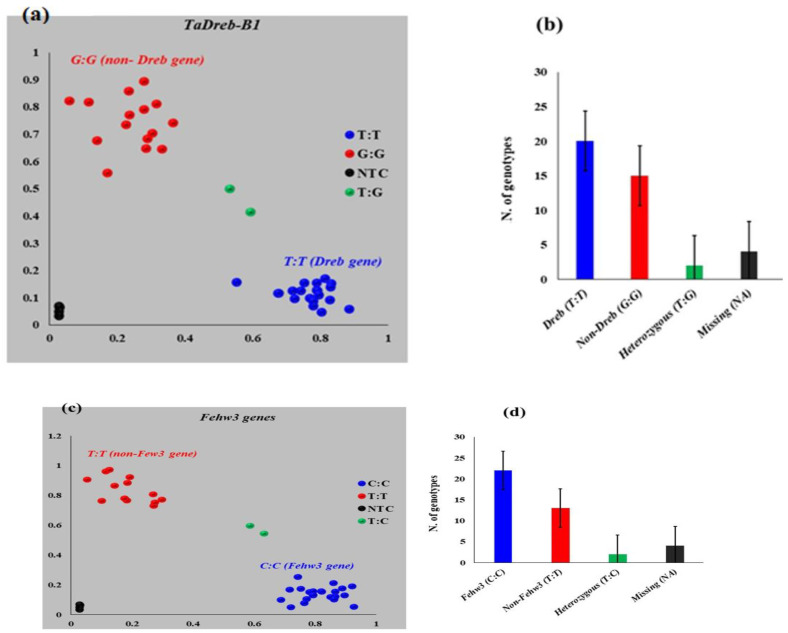
(**a**,**b**). Screening for *TaDreb-B1* using Kompetitive allele-specific PCR (KASP) markers. (**c**,**d**). Screening for *Fehw3* using Kompetitive allele-specific PCR (KASP) markers, where NTC refers to water.

**Figure 4:**
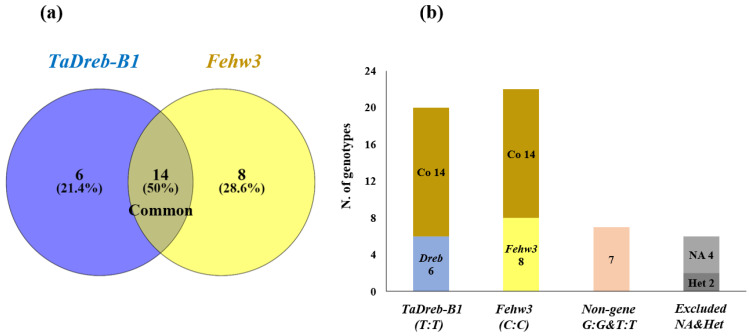
(**a**,**b**). Venn diagram for the common genotypes that contain both types of specific drought genes (*TaDreb-B1*, *Fehw3*), where Co refers to common genotypes that contain the two target genes (*TaDreb-B1*, *Fehw3*), excluded genotypes included both heterozygous loci (*Het*) and missing genotypes (NA).

All genotypes were screened for the presence of *TaDreb-B1* and *Fehw3* genes (Figure 3a–d and Figure 4a,b). The genotypes in each gene were clearly divided into the following three groups, namely homozygous for the gene’s presence, heterozygous, and homozygous for the gene’s absence. A set of 20 genotypes were found to carry the *TaDreb-B1* gene, (Figure 3a,b), while 22 genotypes carried the *Fehw3* gene (Figure 3c,d). A total of 14 genotypes carried both target genes (*Tadreb-B1* and *Fehw3*) detected, and 7 genotypes did not have either of the two genes (Non-gene) (Figure 4a,b). For each gene, a T-test was performed in each trait between the genotypes, with the gene and without the target allele. No significant differences were found between the two groups (gene vs. non-gene) for each trait.

**Figure 5:**
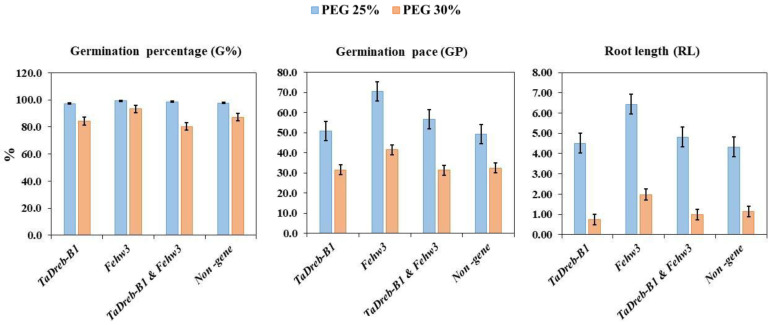

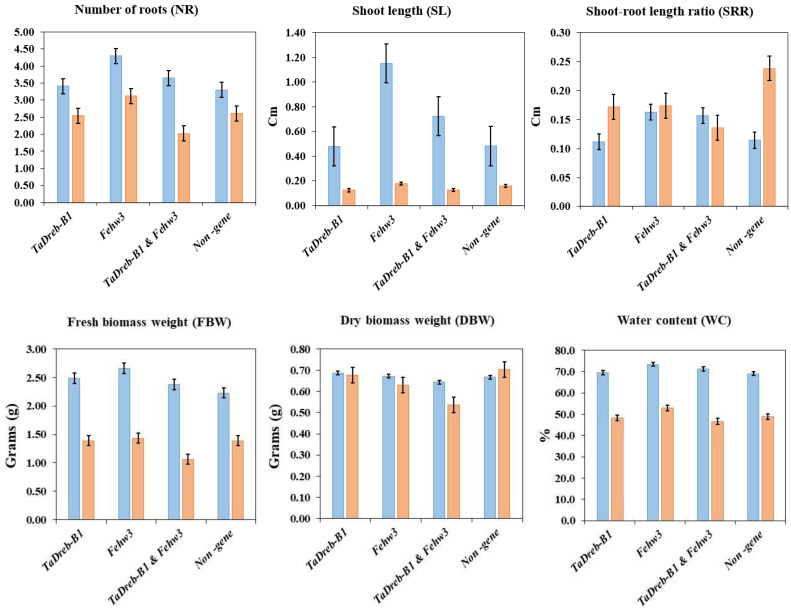
The average of each group for each trait under 25%and 30% of PEG. *TaDreb-B1*: refers to the genotypes with only *TaDreb-B1* allele; *Fehw3*: refers to the genotypes with only the *Fehw3* allele; *TaDreb-B1* and *Fehw3*: refers to those genotypes that having both alleles; Non: refers to the genotypes which had any of the two genes.

To investigate of the two genes efficacy in improving drought tolerance, all genotypes were divided into the following four groups, namely, non-gene, *TaDreb-B1, Fehw3*, and *Fehw3* and *TaDreb-B1*. Then, each trait’s average was scored and compared under two drought stress conditions among the four groups (Supplementary Tables S5 and S6 and Figure 5). Under PEG25%, the genotypes with *Fehw3* had a better performance than the average of the genotypes in the other three groups in all traits except dry biomass weight (DBW). However, under PEG30%, the *Fehw3* group had a better performance than the genotypes’ average performance in the other three groups in all traits except SRR and DBW (Figure 5). A high *Fehw3* effect on GP, RL, NR, and SL under PEG25% was observed. On the other hand, it was observed that there were differences between the *Fehw*3 group and the other groups in G%, GP, RL, and NR under PEG30%. (Supplementary Tables S5 and S6).

## 4. Discussion

### 4.1. Genetic Variation in Drought Tolerance at the Germination Stage 

Evaluation of drought tolerance at the germination stage is critical for crop establishment and its effect on grain yield [29]. In this study, the phenotypic variations in germination and seedling traits among 41 highly diverse spring wheat genotypes under two severe drought stress conditions (PEG25% and PEG30%) were reported. 

The important genetic variation reported for all traits under both PEG treatments as well as the H^2^ estimates (>0.70), are very useful for common wheat breeding programs to efficiently select the drought-tolerant genotypes and improve drought tolerance [12,30,31]. Both parameters are highly important for selecting traits of interest and also for determining which genotypes will perform better than others under drought stress [2,32] and can be used in crossing programs. Effects on germination and seedling traits under PEG-6000 solution has been widely reported using PEG concentrations ranging from 5% to 20% [9,10,14,33]. Nevertheless, studies reporting the effects on these parameters using higher concentrations of >25% of PEG, such as those documented in this work, are scarce. Reductions were reported on SL (−81.1%), RL (−75.9%), FBW (−48.9%), GP (−41.1%), NR (−34.3%), WC (−31.6%), DBW (−15.5%), G% (−13.1%) and SRR (−6.7%) when comparing between PEG30% and PEG25%. Studies reporting on the effect of PEG30% while mimicking a severe drought condition are lacking. Othmani et al. [33] reported detrimental effects on G%, GP, and seedling growth traits (SL, RL, NR, RSR) under six different PEG concentrations (ranging from 5% to 25%) on several durum wheat (*T. durum*) genotypes.

Under PEG25% treatments, the genotypes Citr15314 from Afghanistan and Iwa8600064 from Iran showed a high performance in seven of the traits evaluated, while Sakha-39 (Egypt) and Gn-14 (Canada) presented a poor performance in at least four of the traits (Supplementary Tables S2b and S3b).

High-performance cultivars for PEG30% treatments included Citr15314 (Afghanistan), Iwa8600064 (Iran), Gimmeiza-12 (Egypt), Grekum-105 (Kazakhstan), and Rhodesian Sabanero (PI230202) from Kenya. Sohag-3 (Egypt), Habb (Saudi Arabia), GN_14 (Canada), Lrs-1F193 (Canada), Musane (Oman), CItr15467 (Tunisia), and PI525318 from Morocco presented a low performance in at least four of the traits evaluated (Supplementary Tables S3a,b). The most stable drought-tolerant genotype, Citr15314 (Afghanistan), across the two treatments was characterized as intermediately drought tolerant at the seedling stage [21]. The most tolerant genotypes at the target PEG concentrations are recommended to be re-evaluated at higher concentrations, as a further test, to select the most drought-tolerant genotypes [8]. 

### 4.2. Correlation among Traits

Highly significant and positive associations among the majority of the characters evaluated in both PEG treatments were reported, while SRR and DBW showed weaker associations. In this sense, Othmani et al. [33] reported that SL showed a positive correlation with RL (r = 0.74), SSR (r = 0.46) and NR (r = 0.67). Rauf et al. [34] reported a significant and positive correlation between the germination rate, coleoptile length, SL, and RL but found a non-significant and negative correlation between the germination rate and SSR in several wheat cultivars. Moreover, Khan et al. [35] reported that RL exhibited a positive and significant correlation with coleoptile length, fresh shoot weight, and dry shoot weight. Sharma et al. [14] evaluated 15 Indian wheat genotypes and also reported a positive correlation among the G% and seedling growth (RL and SL) parameters under 5%, 10%, and 15% PEG concentrations. Our results showed that the root growth traits (RL and NR) presented a higher positive correlation with other germination and seedling growth parameters, which is in agreement with the study by Ahmed et al. [36], who also documented a similar response when evaluating 105 diverse wheat genotypes. In general terms, higher correlations were reported under severe drought (PEG30%) treatments for GP, G%, NR, RL, SL, FBW, and WC compared to PEG25%. Some discrepancies between the correlations among the evaluated characteristics were detected under both treatments of PEG. In concordance with our results, Sharma et al. [14] also reported higher correlations between the G% with SL and RL under increased PEG concentrations. The DTI, scored at the seedling stage by Ahmed et al. [21], did not correlate with any of the germination traits under either treatment, except for FBW and DBW. Little to no correlation in drought tolerance between the germination and the seedling stage was reported by Hasseb et al. [23] and Moursi et al. [2]. Therefore, it is very important to test the same genotypes across different growth stages.

### 4.3. Fehw3 and TaDreb-B1 KASP Markers for Improving Drought Tolerance at the Germination Stage 

DNA molecular markers used for genotyping, such as SSR, RFLP and AFLP, etc, screen elite genotypes for the presence or absence of target genes and/or QTLs. However, all of these DNA markers require gel electrophoresis which, may require specific precautions when using unsafe chemicals, such as ethidium bromide. Moreover, these DNA markers also require high-resolution melting to separate products, which are low throughput, high cost, and labor-intensive [10,17]. Instead, KASP technology has high reproducibility, precise genotyping, and affordability [34]. Two KASP markers for *TaDreb-B1* [16] and *Fehw3* [4] were used in this study to screen the elite genotypes for the presence of two genes and study their effect on the germination traits under severe drought stress. SNP discovery and validation is an important step in marker-assisted selection in wheat due to its polyploidy nature, large genome size, and highly repetitive sequence [37]. Therefore, validation on newly developed SNP markers, which are associated with target trials should be performed in various ways. In a study by Rehman et al. [38], two KASP markers were used to screen the presence or absence of the *Fehw3* and *TaDreb-B1* genes in a set of 200 wheat genotypes. They studied the diversity of these markers in the elite genotypes and reported that the favorable allelic variation was significantly associated with grain yield-contributing traits. In this study, two KASP markers in a different genetic background were tested under two drought stress conditions at the germination stage. Moreover, the two genes’ effect when combined or alone under severe drought tolerance were investigated. Therefore, the response of *TaDreb-B1* and *Fehw3* on germination growth parameters under high PEG-6000 concentrations has not been previously documented and is the main novelty reported in this work.

The results of the genotyping revealed three distinguishing clusters among the homozygous for favorable allele, heterozygous loci, and homozygous for unfavorable alleles, in each gene. These clusters, found in each, indicate the reality of the two KASP markers and show that the primer had a high specificity for detecting the presence or absence of the gene. 

The KASP technology detected 14 genotypes carrying both the *TaDreb-B1* and *Fehw3* genes. In this study, no significant association (*p* < 0.01) was found between the two contrasting groups (allele vs non-allele) for each gene (Supplementary Table S7). On average, it was that the genotypes with only *Fehw3* showed a high performance compared to the other groups under both treatments. This effect was observed in GP, RL, NR, SL, FBW, and WC, and under both treatments. Therefore, *Fehw3* might be more important than the *TaDreb-B1* gene in improving germination traits under different drought stress treatments.

Unfortunately, few studies have focused on *Fehw3* and its relation to drought tolerance compared to the *TaDreb-B1* gene. Therefore, the association of this marker with drought tolerance should be confirmed in further studies. Remarkably, it was observed that the genotypes with neither gene performed better compared to those with either gene alone in many traits, especially under PEG30%. This could be due to the fact that drought tolerance is a polygenic trait controlled by many genes. Thus, the non-genes group may have other more important drought-tolerant genes instead of *Fehw3* and *TaDreb-B1.* The effect of two KASP markers, *TaDreb-B1* and *Fehw3* on germination traits under two different drought treatments was not significant. 

Under field conditions, the *1-Fehw3* gene was found to contribute to the high stem water-soluble carbohydrate (WSC) remobilization under drought stress [4]. The cleaved amplified polymorphic (CAP) marker of *1-Fehw3* was reported as a useful marker for selecting high stem WSC remobilization and high thousand-grain in wheat breeding under terminal drought conditions [4]. In this study, *Fehw3* alone was found to improve all traits under PEG30% compared to *TaDreb-B1*. Therefore, the same gene might play an important role in improving drought tolerance during wheat’s adult growth stage than early growth stages. *Dreb* genes have been widely used in molecular breeding programs to improve wheat’s drought tolerance [8]. *Dreb* proteins include a large family of transcription factors that play a vital role in regulating some important genes closely related to drought [39]. 

## 5. Conclusions

This study investigated the performance of the elite genotype which represented tolerance, intermediate drought tolerance, and susceptibility to drought stress at the seedling stage [21]. Not all tolerant genotypes at the seedling stage provided high performance for germination under 25% and 30% of PEG, while some susceptible genotypes to drought at the seedling stage showed good germination under drought stress. Therefore, it is very important to investigate drought tolerance at different growth stages. The two markers divided the genotypes into clear clusters, indicating the accuracy of genotyping and determining the precise absence or presence of the target alleles. For each gene, no significant differences were found between genotypes that had the gene and those without the gene. Further studies should be conducted to test the two KASP markers for improving drought tolerance in wheat. The Citr15314 genotype showed a stable performance for most of the traits under both drought stress treatments (different concentrations of PEG (25% and 30%). This genotype (Citr15314), an intermediate drought tolerant at the seedling stage carried *Fehw3* allele. Therefore, this genotype could be included as a parent in future breeding programs to improve drought tolerance during the early growth stage. 

## Figures and Tables

**Table 1:** Basic information on the KASP primer sequences used in this study.

*Gene*.	Chr.	Allele	Phenotype	Forward Primer	Reverse Primer	Reference
*TaDreb-B1*	3BS	*Dreb-B1a*	Tolerant	GAAGGTGACCAAGTTCATGCTCCTGCGCACTTTCTTCTTCCTGT	TTTCACCTTGTGATATGGATTGCCTTGAT	Wei et al. [16]
		*Dreb-B1b*	Susceptible	GAAGGTCGGAGTCAACGGATTCTGCGCACTTTCTTCTTCCTGG		
1-*Fehw3*	6BS	Westonia type	High expression	GAAGGTGACCAAGTTCATGCTCTCCCCCCTTCCTTCTGTCC	AGGAAGACGGCCCGAGCTTT	Zhang et al. [4]
		Kauz type	Low expression	GAAGGTCGGAGTCAACGGATTCTCCCCCCTTCCTTCTGTCT		

Where; *TaDreb-B1* refers to dehydration responsive element binding proteins, while *1-Fehw3* refers to fructan 1-exohydrolase w3.

**Table 2:** Analysis of variance (ANOVA) for the seedling traits scored on 41 spring wheat genotypes under two PEG concentrations (25% and 30%).

Source of Variance	GP	G%	NR	SL	RL	SRR	FBW	DBW	WC
Treatments (concentration 25%, 30%)	2111.60 **	580.81 **	980.07 **	842.35 **	3029.21 **	4.65 *	1427.63 **	136.83 **	797.67 **
Replications (R)	7.28 **	1.04 ns	1.06 ns	0.16 ns	2.23 ns	0.2 ns	0.48 ns	5.06 **	0.72 ns
Genotypes (G)	41.75 **	25.07 **	53.27 **	23.89 **	28.81 **	23.76 **	26.48 **	47.25 **	8.26 **
Treatment (T) × Genotypes(G)	15.15 **	19.22 **	16.99 **	20.12 **	16.70 **	32.70 **	12.58 **	26.66 **	6.17 **
Heritability (H^2^)	97.6	96.01	98.12	95.81	96.53	95.79	96.22	97.88	87.9

Germination pace (GP); Germination percentage (G%); Number of root (NR); Shoot length (SL); Root length (RL); Shoot-root length ratio (SRR); Fresh biomass weight (FBW); Dry biomass weight (DBW); Water content (WC). *, ** significant at *p* ≤ 0.05 and *p* ≤ 0.01, respectively. (ns) refers to non-significant.

**Table 3:** Minimum (Min), maximum (Max), mean, least significant differences (LSD), F-value (among genotypes), coefficient of variation (CV), phenotypic standard deviation (SD), and broad- sense heritability (H^2^) under two PEG-6000 concentrations on 41 spring wheat genotypes.

Tait	Concentration	Min	Max	Mean	CV	*SD*	LSD	*F-Value*	*H^2^*
Germination percentage (G%)	25%	86.67	100	98.39	3.25	3.19	2.93	9.41 **	89.4
	30%	45	100	85.23	18.64	8.94	10.01	19.94 **	94.9
germination pace (GP)	25%	34.87	97.62	57.65	26.19	15.09	8.15	27.19 **	96.3
	30%	19.58	66.74	33.93	26.35	15.89	4.53	30.89 **	96.8
Number of roots (NR)	25%	1.27	5.67	3.62	24.53	0.89	0.63	15.85 **	93.7
	30%	0.78	5	2.38	47.48	1.13	0.32	79.92 **	98.7
Shoot length (SL)	25%	0.18	2.82	0.74	82.68	0.61	0.37	21.71 **	95.4
	30%	0	0.41	0.14	73.10	0.10	0.07	16.52 **	93.9
Root length (RL)	25%	1.93	8.17	4.98	35.41	1.76	1.13	19.21 **	94.8
	30%	0.16	3.81	1.20	81.88	0.98	0.51	29.13 **	96.6
Shoot/root length ratio (SRR)	25%	0.04	0.39	0.15	61.50	0.90	0.06	18.16 **	94.5
	30%	0	0.68	0.14	103.89	0.16	0.07	3.43 **	70.8
Fresh biomass weight (FBW)	25%	1.29	4.14	2.39	31.42	0.75	0.48	19.40 **	94.8
	30%	0.6	2.15	1.22	34.38	0.42	0.3	15.37 **	93.5
Dry biomass weight (DBW)	25%	0.42	2.67	0.71	48.74	0.34	0.1	87.50 **	98.9
	30%	0.38	0.94	0.6	22.80	0.14	0.14	8.06 **	87.6
Water content (WC)	25%	38.51	84.03	69.63	11.86	8.26	6.66	12.20 **	91.8
	30%	32.52	67.24	47.65	21.31	10.15	12.74	5.03 **	80.1

** significant at the 0.01 level of probability.

**Table 4:** Correlation between the germination traits under both treatments (PEG25% and PEG30%) with the drought tolerance index (DTI) at the seedling stage; taken from Ahmed et al. [21].

Traits	Concentration PEG25%	Concentration PEG30%
**GP**	0.244	−0.171
**G%**	0.067	−0.275
**NR**	0.238	−0.087
**RL**	0.289	−0.249
**SL**	0.256	−0.308
**SRR**	0.001	−0.123
**FBW**	−0.350 *	−0.396 *
**DBW**	−0.549 **	−0.406 **
**WC**	0.187	−0.155

Germination pace (GP); Germination percentage (G%); Number of root (NR); Root length (RL); Shoot length (SL); Shoot-root length ratio (SRR); Fresh biomass weight (FBW); Dry biomass weight (DBW); Water content (WC). *, ** significant at *p* ≤ 0.05 and *p* ≤ 0.01, respectively.

## Data Availability

The sequence data presented in this study are not publicly available due to some ongoing projects on the same plant materials. Other data is presented in the supplementary files.

## Supplementary Materials

The following supporting information can be downloaded at: https://www.mdpi.com/article/10.3390/genes14020373/s1, Supplementary Table S1: Detailed information of the 41 highly diverse spring wheat genotypes used in this study; name, and country of each genotype. Supplementary Table S2a: Phenotypic scores for each tested genotype in germination traits scored under PEG25%. Supplementary Table S2b: List of high and low performances genotypes under PEG25%. Supplementary Table S3a: Phenotypic scores for each tested genotype in the germination traits scored under PEG30%. Supplementary Table S3b: List of high and low performances genotypes under PEG30%. Supplementary Table S4: Reduction in each germination traits due to severe drought stress (PEG30%). Supplementary Table S5: Phenotypic scores for all genotypes under PEG25% in the four groups classified based on the presence or absence of the two genes. Supplementary Table S6: Phenotypic scores for all genotypes under PEG30% in the four groups classified based on the presence or absence of the two genes. Supplementary Table S7: Marker- traits association analysis between the two KASP marker (*Tadreb-B1* and *1-Fehw3)* and germination traits under both concentrations of PEG (25% and 30%). Supplementary Figure S1: The number of genotypes used from each country and the names of the continents to which these countries belong. Supplementary Figure S2: (a)The phenotypic variation among genotypes under both concentrations of PEG (25% and 30%), (b) phenotypic variation between susceptible and tolerant genotyped under PEG25%, (c) phenotypic variation between susceptible and tolerant genotypes under PEG30%. Supplementary Figure S3: Phenotypic correlation in (a) germination percentage, (b) germination pace, (c) root length, (d) number of roots length, (e) shoot length, (f) shoot-root length/ ratio, (g) fresh biomass weight, (h) dry biomass weight, (i) water content traits under both concentration of PEG25 % and 30 %.

## Abbreviations

WCC	Wheat Core Collection
PEG-6000	Polyethylene Glycol
G%	Germination Percentage
GP	Germination Pace
NR	Number of Roots
SL	Shoot Length
RL	Root Length
SRR	Shoot–Root Length Ratio
FBW	Fresh Biomass Weight
DBW	Dry Biomass Wight
DTI	Drought tolerance index
WC	Water content
H^2^	Heritability
*TaDreb-B1*	Dehydration Responsive Element Binding Proteins
*1-Fehw3*	Fructan 1-exohydrolase w3
PCR	Polymerase Chain Reaction
KASP	Kompetitive Allele Specific PCR
SNP	Single nucleotide polymorphism
