# Supplementary material for: Screening Spring Wheat Genotypes for TaDreb-B1 and Fehw3 Genes under Severe Drought Stress at the Germination Stage Using KASP Technology
Source: Genes (Basel). 2023 Jan 31;14(2):373. doi: 10.3390/genes14020373 (PMC9957104; doi:10.3390/genes14020373)
Supplement: Supplementary file 1 [file genes-14-00373-s001.zip › genes-2119989-supplementary/Supplementaly figures - proof.pptx]

## Slide 1
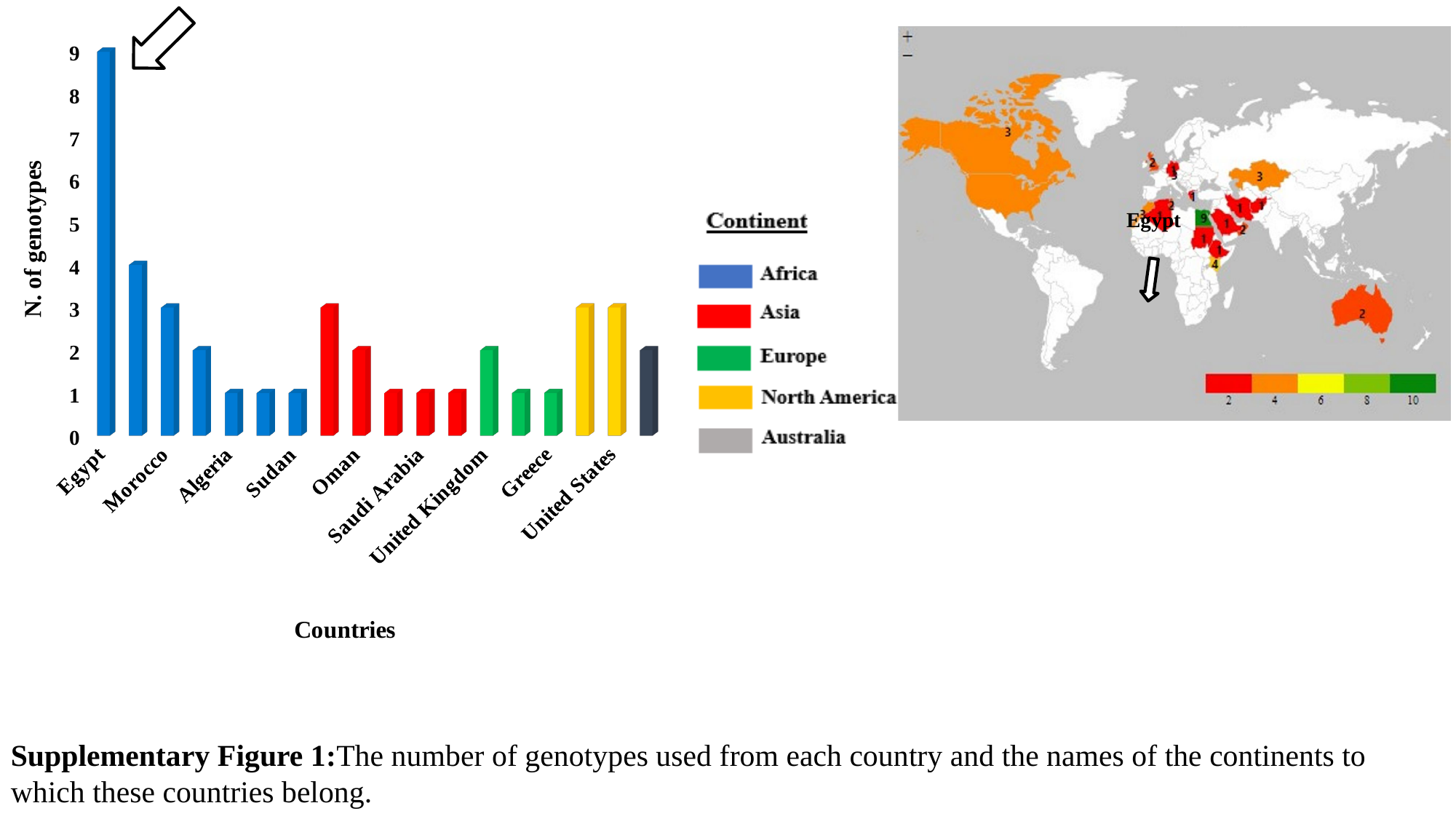

[unsupported chart]
Egypt
Supplementary Figure 1:The number of genotypes used from each country and the names of the continents to which these countries belong.

## Slide 2
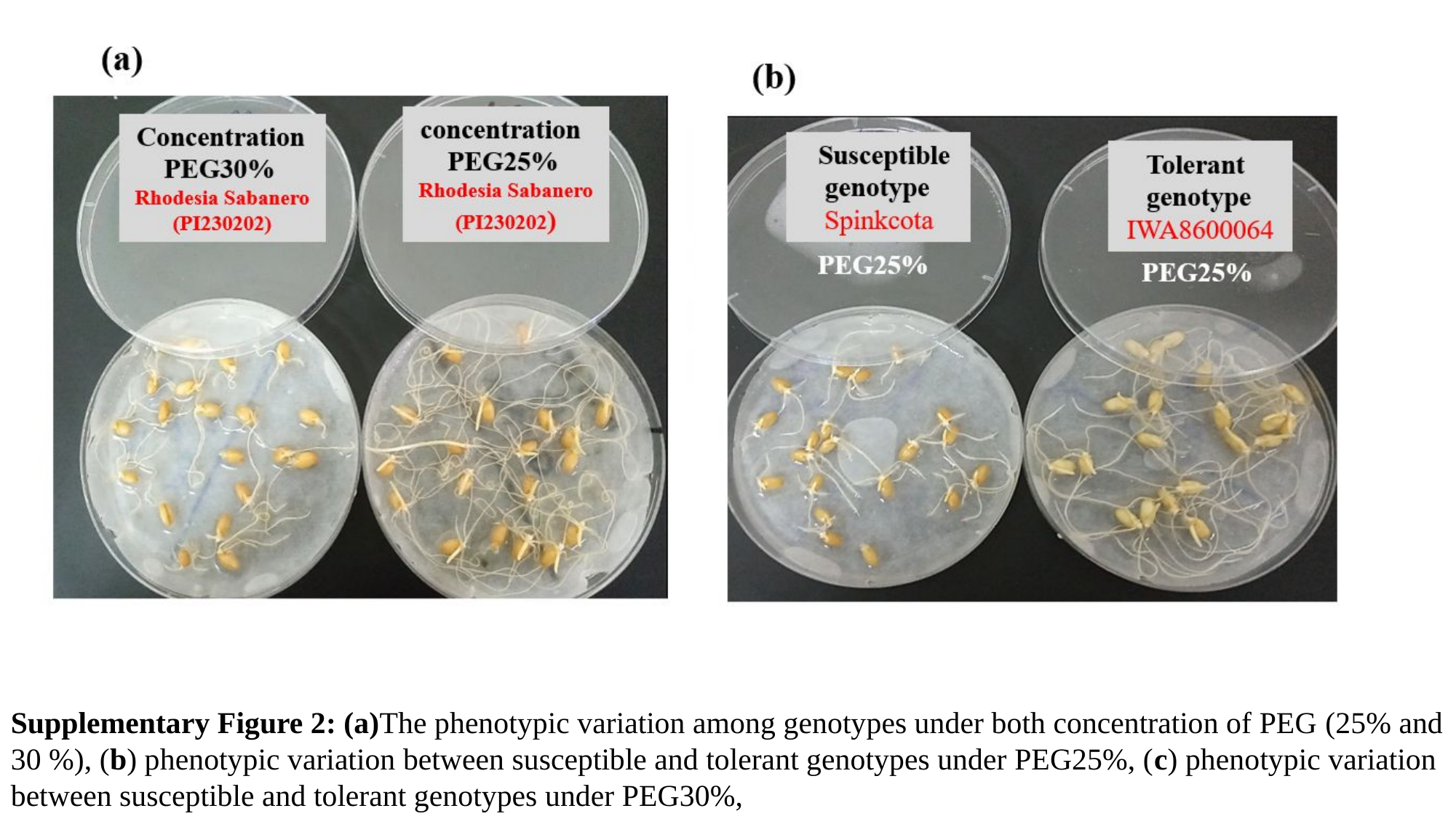

Supplementary Figure 2: (a)The phenotypic variation among genotypes under both concentration of PEG (25% and 30 %), (b) phenotypic variation between susceptible and tolerant genotypes under PEG25%, (c) phenotypic variation between susceptible and tolerant genotypes under PEG30%,

## Slide 3
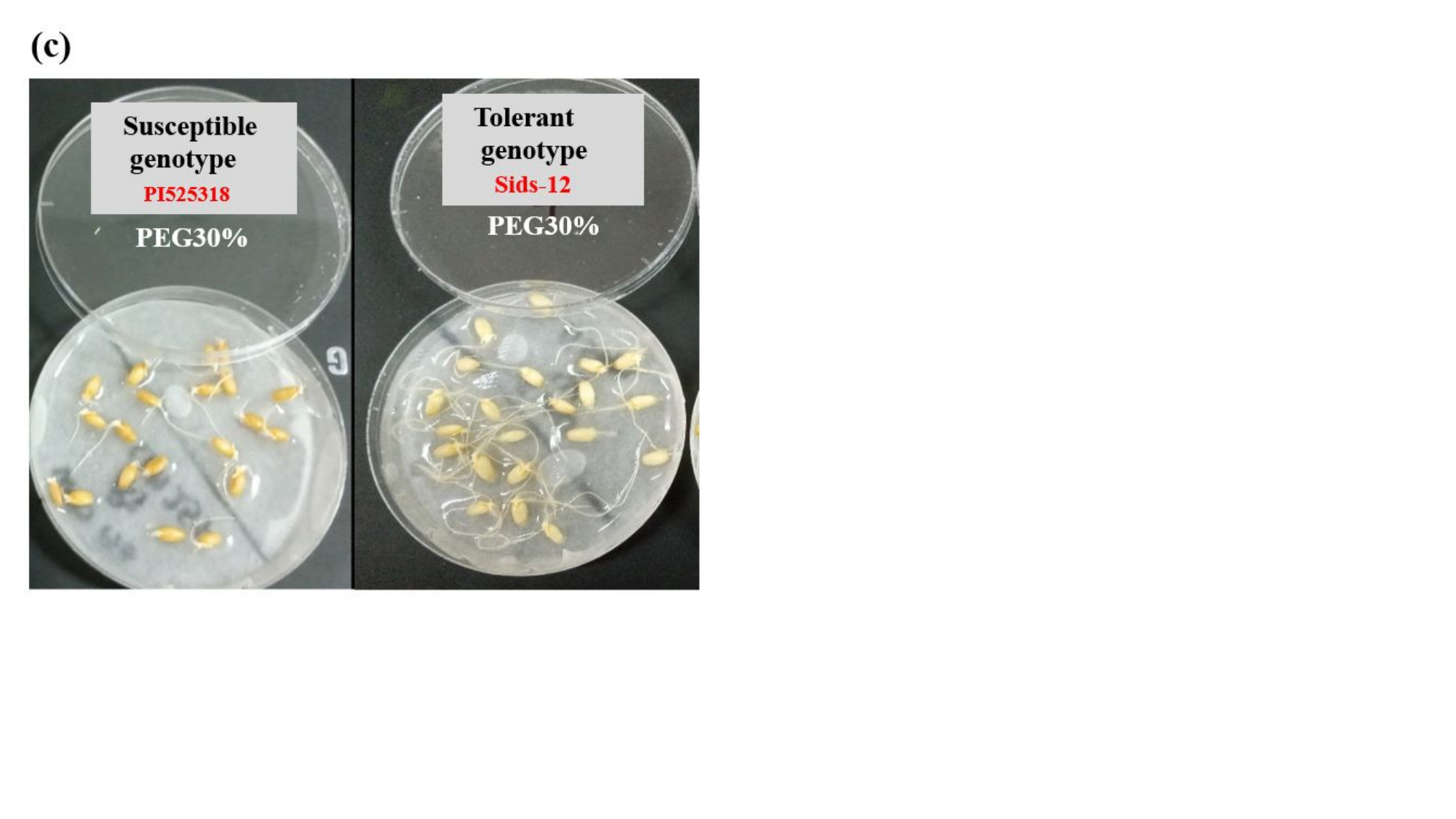

## Slide 4
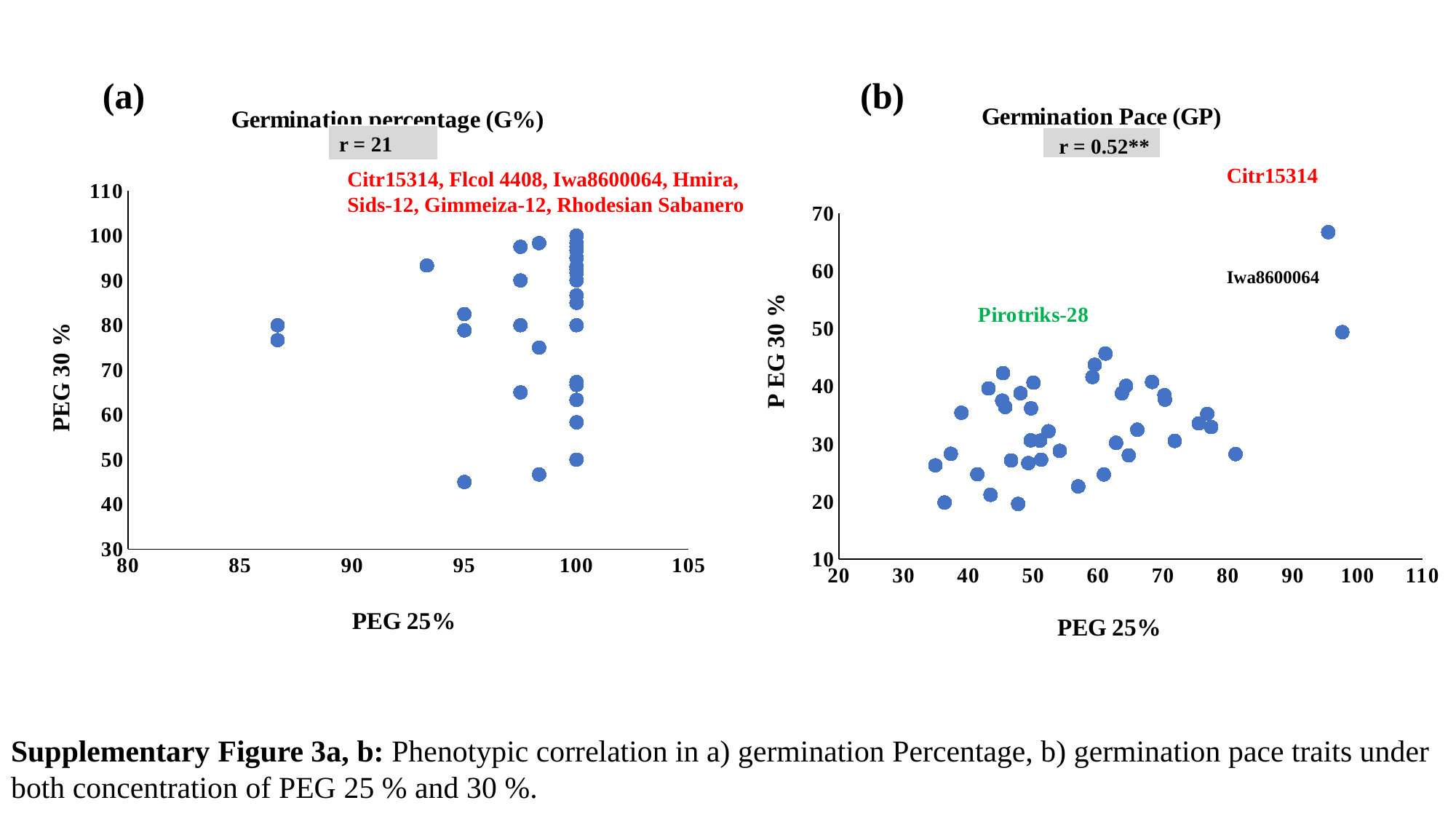

(a)
(b)
### Chart: Germination Pace (GP)
| Category | |
|---|---|
### Chart: Germination percentage (G%)
| Category | |
|---|---|r = 21
 r = 0.52**
Citr15314
Citr15314, Flcol 4408, Iwa8600064, Hmira, Sids-12, Gimmeiza-12, Rhodesian Sabanero
Iwa8600064
Supplementary Figure 3a, b: Phenotypic correlation in a) germination Percentage, b) germination pace traits under both concentration of PEG 25 % and 30 %.

## Slide 5
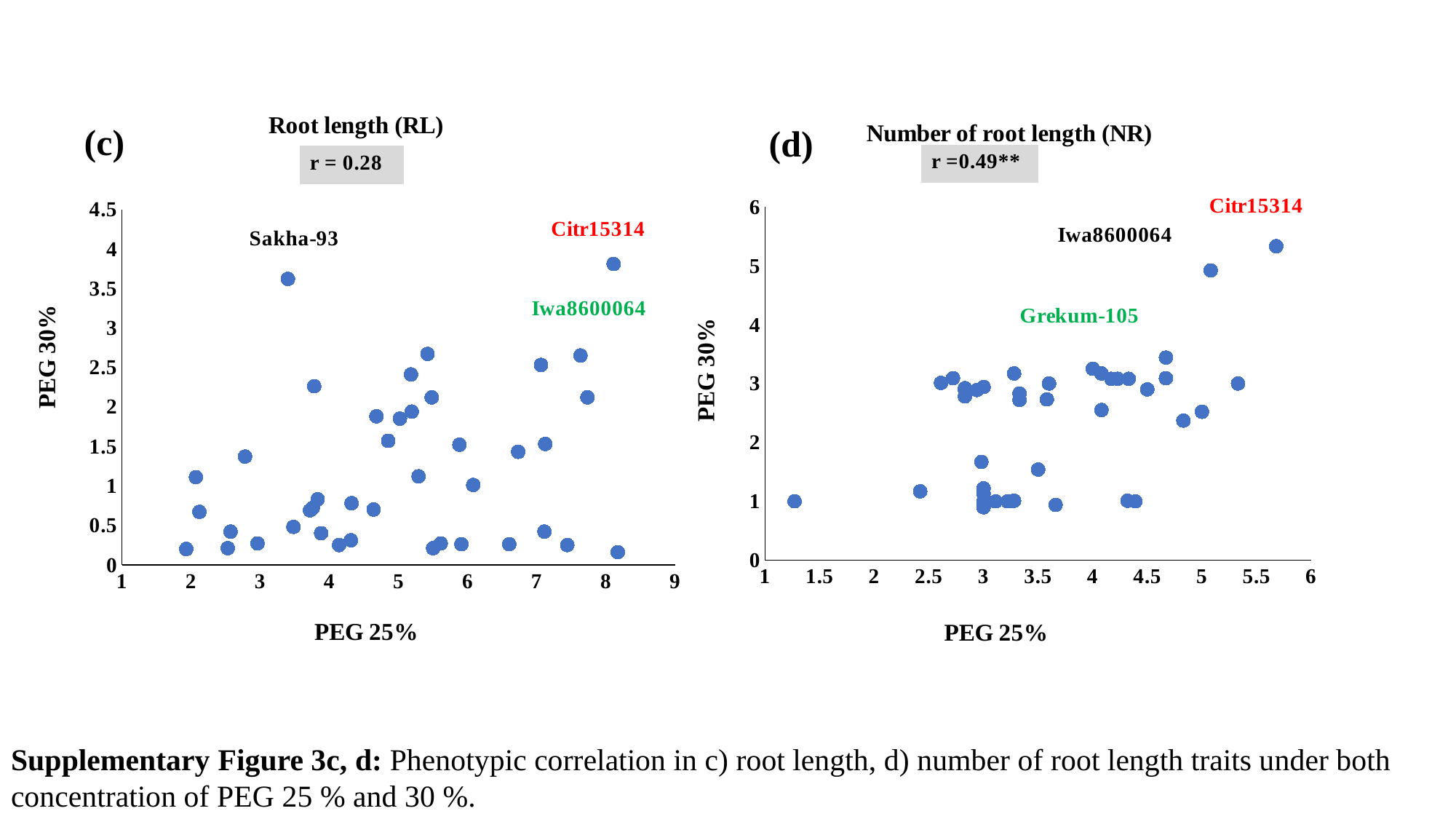

### Chart: Root length (RL)
| Category | |
|---|---|
### Chart: Number of root length (NR)
| Category | |
|---|---|(c)
(d)
Supplementary Figure 3c, d: Phenotypic correlation in c) root length, d) number of root length traits under both concentration of PEG 25 % and 30 %.

## Slide 6
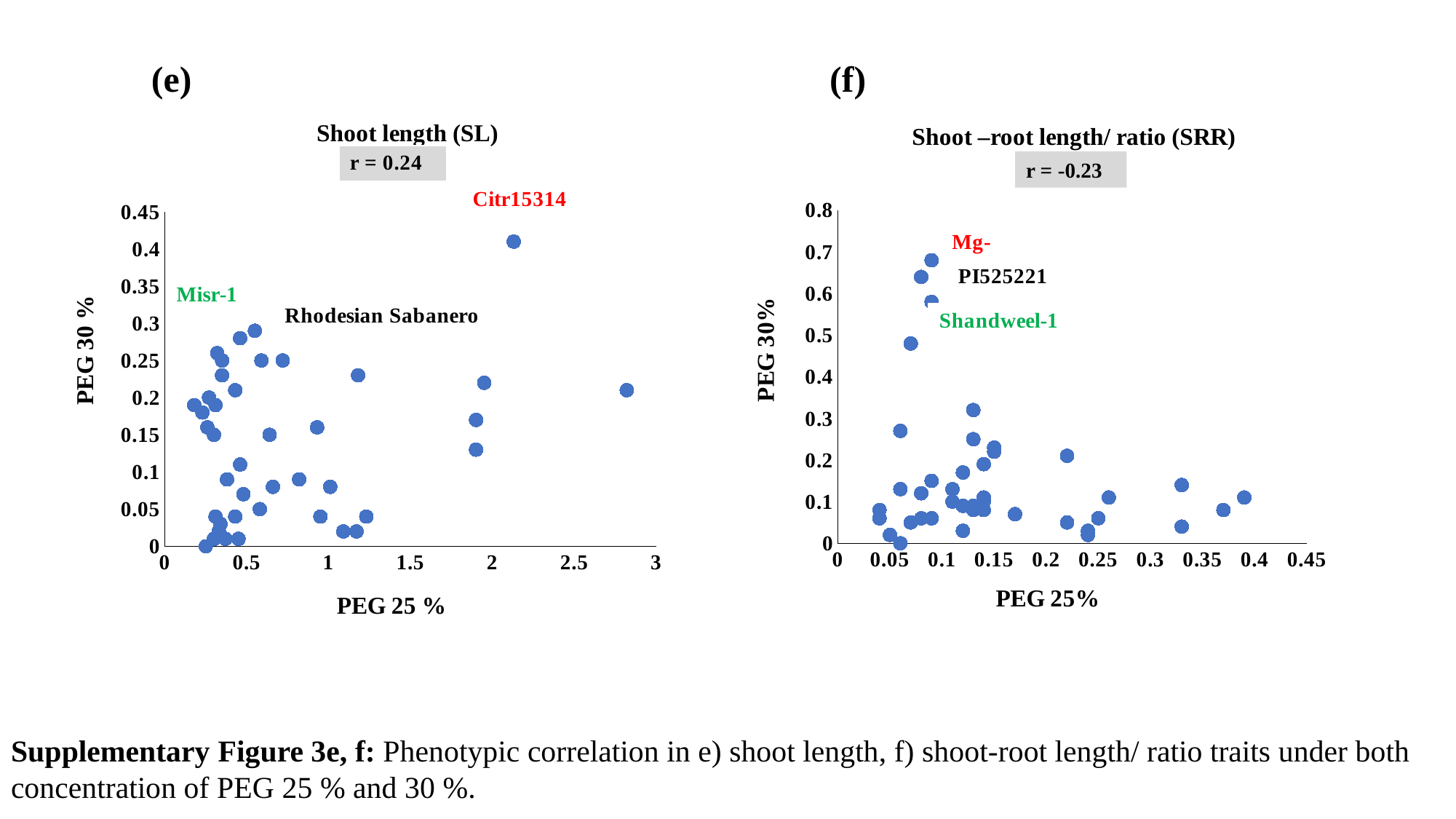

(e)
(f)
### Chart: Shoot –root length/ ratio (SRR)
| Category | |
|---|---|
### Chart: Shoot length (SL)
| Category | |
|---|---|r = -0.23
Supplementary Figure 3e, f: Phenotypic correlation in e) shoot length, f) shoot-root length/ ratio traits under both concentration of PEG 25 % and 30 %.

## Slide 7
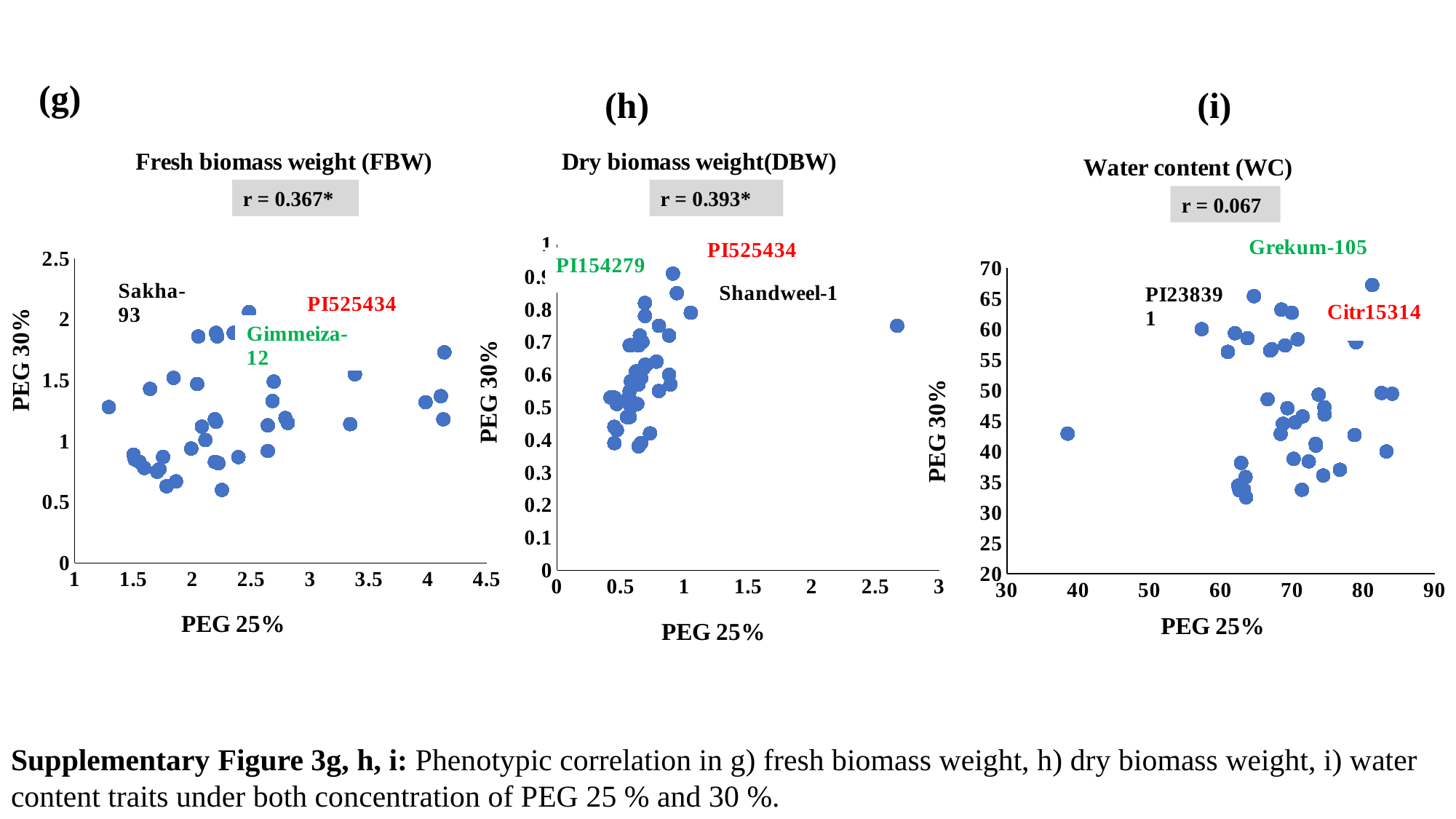

(g)
(i)
(h)
### Chart: Dry biomass weight(DBW)
| Category | |
|---|---|
### Chart: Water content (WC)
| Category | |
|---|---|
### Chart: Fresh biomass weight (FBW)
| Category | |
|---|---|r = 0.367*
r = 0.393*
r = 0.067
Supplementary Figure 3g, h, i: Phenotypic correlation in g) fresh biomass weight, h) dry biomass weight, i) water content traits under both concentration of PEG 25 % and 30 %.
